# Poor sleep and high rheumatoid arthritis risk: Evidence from large UK Biobank cohort

**DOI:** 10.1371/journal.pone.0318728

**Published:** 2025-04-23

**Authors:** Yi-Qun Yang, Han-Wen Cao, Xing-Hao Yu, Lin Bo, Fei-Yan Deng, Shu-Feng Lei

**Affiliations:** 1 Collaborative Innovation Center for Bone and Immunology between Sihong Hospital and Soochow University; Center for Genetic Epidemiology and Genomics, School of Public Health, Suzhou Medical College of Soochow University, Suzhou, Jiangsu, P. R. China; 2 Jiangsu Key Laboratory of Preventive and Translational Medicine for Major Chronic Non-Communicable Diseases, Soochow University, Suzhou, Jiangsu, P. R. China; 3 National Clinical Research Center for Hematologic Diseases, Jiangsu Institute of Hematology, The First Affiliated Hospital of Soochow University, Suzhou, Jiangsu, P. R. China; 4 Department of Rheumatology, The Second Affiliated Hospital of Soochow University, Suzhou, Jiangsu, P. R. China; The First Affiliated Hospital of Soochow University, CHINA

## Abstract

**Objective:**

To evaluate the relationship between sleep behaviors and rheumatoid arthritis (RA) risk.

**Methods:**

First, based on large-scale data from the UK Biobank, we analyzed the associations between multiple sleep factors and RA risk and calculated a poor sleep score (PSS) to assess their combined effect. Then we constructed genetic risk scores (GRS) based on a large genome-wide association study and assessed the interaction or combined effect of sleep and genetic factors on RA risk. Finally, we conducted a case-control study to explore the effect of RA on sleep.

**Results:**

Sleep duration, getting up, napping during the day, insomnia, and daytime dozing were associated with the risk of RA, but no associations were observed for chronotype and snoring with RA. Participants in the high genetic risk and high PSS group had a 363.1% higher risk of developing RA compared to those with low genetic risk and low PSS. We also found that women were more likely than men to be affected by the combined effects of PSS and GRS. In the case-control study, there were statistically significant associations of RA with getting up, PSS grade and PSS.

**Conclusions:**

Unhealthy sleep patterns were associated with increasing risk of RA among participants with low, intermediate, or high genetic risk.

## Introduction

Rheumatoid arthritis (RA) is a chronic systemic inflammatory disease characterized by synovitis. If left untreated, it can lead to joint deformity and loss of function. The pathogenesis of RA is still under investigation, but it may involve a combination of autoimmune factors, genetics, smoking, and other environmental influences [1]. Individuals with RA often experience pain, obesity, and poor sleep, which significantly reduce their quality of life compared to those without the condition [2,3].

Sleep is essential for maintaining human health [4,5]. While the relationship between RA and sleep has been extensively studied, it remains unclear whether poor sleep is a cause or consequence of RA. In most cases, sleep disturbances in RA patients are linked to pain. A Swedish case-control study found that sleep problems worsen as the duration of the disease is extended [6]. A cross-sectional, multicenter study involving 305 Belgian patients reported that poor sleep quality is associated with inadequate RA control, potentially due to pain [5]. Moreover, medications prescribed for RA treatment may further impact sleep quality [5,7,8]. Recent research has highlighted a strong connection between sleep and the immune system, which plays a crucial role in complex diseases [9]. During sleep, the immune system releases proteins called cytokines, some of which promote sleep. In the presence of infection or inflammation, the demand for certain cytokines increases [10]. Conversely, sleep deprivation may reduce the production of these protective cytokines [9], leaving the body with fewer antibodies and cells to combat infection [11].

The relationship between poor sleep and various complex diseases has been a focus of recent investigation. Studies have shown that women with extreme sleep durations are more likely to develop metabolic syndrome and exhibit higher metabolic syndrome severity scores. In contrast, women who consistently sleep for seven hours per night have the lowest severity scores for metabolic syndrome [12]. A meta-analysis revealed a U-shaped relationship between sleep duration and the risk of osteoporosis, with the lowest risk observed among individuals who slept for eight hours per night [13]. Additionally, a UK cohort study demonstrated that subjects who slept fewer than four hours or more than ten hours per night experienced significantly faster cognitive decline compared to those who slept seven hours per night [14]. Either too much or too little sleep increased all-cause mortality and cardiovascular events, with 7 hours of sleep per night having the lowest risk [15]. Several studies have also suggested that poor sleep might contribute to the development of RA [16–18]. However, most of these studies are limited by cross-sectional designs and small sample sizes, and have only established a correlation between sleep and RA. There is a lack of research on whether sleep characteristics modulate the relationship between genetic factors and RA. Importantly, the interaction between sleep characteristics and genetic factors in determining RA risk remains largely unknown.

A composite measure of sleep health, known as the sleep score, was recently proposed. A prospective study examined the combined association of five sleep behaviors—sleep duration, chronotype, insomnia, snoring, and excessive daytime sleepiness—in the form of a healthy sleep score, with the risk of cardiovascular disease (CVD) [19].

**Fig 1:**
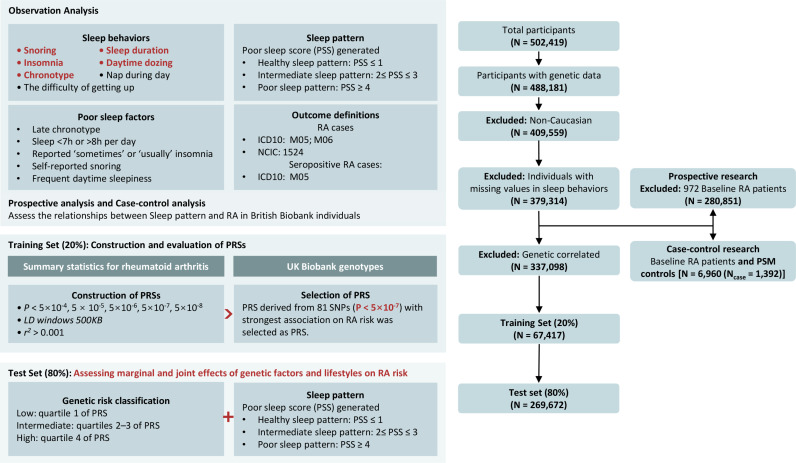
Overflow of our research.

Building on this concept, we prospectively assessed the effects of seven sleep behaviors on the risk of developing RA. To better understand the combined effect of poor sleep patterns and high genetic risk on RA, we followed [19] and constructed a poor sleep score (PSS) based on key sleep behaviors. Additionally, we examined the mediation effect of inflammation as a potential pathway from PSS to RA incidence. Furthermore, we explored the combined influence of sleep characteristics and genetic susceptibility on RA risk, and investigated potential gene-environment interactions. Finally, we conducted a case-control study using baseline data to investigate the impact of RA on sleep. The findings of our research are illustrated in Fig 1.

## Materials and methods

### Study design

We first examined the associations between multiple sleep behaviors and RA risk in a prospective study, calculating the PSS for each participant to evaluate its effect on RA risk. The participants were then stratified based on their calculated sleep scores and Genetic Risk Scores (GRS) to investigate the interaction and combined effects of sleep and genetic factors on RA risk. Additionally, a case-control study was conducted to assess the impact of RA onset on sleep quality in later life. Finally we used linkage disequilibrium scores regression (LDSC) [20] to assess the genetic association between sleep behaviors and RA.

### UK Biobank data sets

The UK Biobank is a large biomedical database initiated in 2006, containing comprehensive genetic and health data from 500,000 UK participants aged 40 to 70 (http://www.ukbiobank.ac.uk/). The study has collected, and continues to collect, detailed genotypic and phenotypic information, including questionnaires, physical measurements, multimodal imaging, and data for a wide range of health-related longitudinal follow-up investigations [21]. Participants from 22 assessment centers with each eligible participant completing a written informed consent form were included at baseline. This database offers extensive health-related information, including biological measurements, lifestyle indicators, and biomarkers in blood and urine. It provides researchers with a unique opportunity to explore the combined effects of genes and the environment on complex diseases, offering a novel approach to understanding disease development. The present analyses were conducted under UK Biobank application number 76875. Detailed information is as follows:

#### Ethics approval statement.

Ethical approval was granted by the Northwest Centre for Research Ethics Committee, and informed consent was obtained from all participants.

#### Definition of RA cases.

Rheumatoid arthritis (RA) was defined according to the International Classification of Diseases, Tenth Revision (ICD-10-CM), as recorded in the UK Biobank (coded as “M05” and “M06”), with seropositive RA defined by “M05.” The outcome event was determined by the onset of RA from baseline to the date of diagnosis, death, loss to follow-up, or January 1, 2022, whichever occurred first. Self-reported non-cancer illness (coded as “1524”) was used to identify RA cases at baseline, which were included in case-control studies and excluded from prospective studies.

#### Definition of sleeping condition.

The data on sleeping habits of the participants was obtained through detailed self-reported questionnaires collected by the UK Biobank. A total of seven sleeping phenotypes were included in our study: sleep duration (Field ID: 1160), difficulty getting up (Field ID: 1170), chronotype (Field ID: 1180), nap during the day (Field ID: 1190), sleeplessness/insomnia (Field ID: 1200), snoring (Field ID: 1210), daytime dozing/sleeping (Field ID: 1220). The definitions of these sleep traits are provided in S1 Table. Poor sleep factors were defined as follows: late chronotype (‘evening’ or ‘evening than morning’); sleep less than 7h or more than 8h per day; reported ‘sometimes’ or ‘usually’ insomnia symptoms; self-reported snoring; and frequent daytime sleepiness (‘usually’). The PSS was generated, incorporating five sleep factors (chronotype, duration, insomnia, snoring, and excessive daytime sleepiness) to better assess an individual’s sleep status and to accurately classify the population. The PSS ranges from 0 to 5, with higher scores indicating a poorer sleep pattern. Sleep patterns were categorized as ‘healthy sleep pattern’ (PSS ≤ 1), ‘intermediate sleep pattern’ (2 ≤ PSS ≤ 3), and ‘poor sleep pattern’ (PSS ≥ 4) based on the constructed PSS. Additionally, a weighted PSS (PSS_CV_) was created using a 10-fold cross-validation procedure as a sensitivity analysis. The weighted PSS was derived by combining the five sleep factors.

### Summary-level data and construction of genetic risk score (GRS)

Genetic data on sleep behaviors (sleep duration [22], difficulty getting up [23], chronotype [24], nap during day [25], insomnia [26], snoring [27], daytime dozing [28]) were utilized in the largest relevant genome-wide study of its kind to date.

In the genetic analysis, we randomly divided the total sample into a training set (20%) and a test set (80%). The training set was used to identify the best-performing GRS, and all subsequent analyses were conducted using the test set. The GRS was calculated based on genotypes of selected single nucleotide polymorphisms (SNP) from UK Biobank under different thresholds (*P* from 5 × 10^−8^ to 5 × 10^−4^), where LD *r*^2^ was set to 0.001 within 1Mb physical distance. The *r*^2^ was derived from the 1000 Genomes Projects for individuals of European ancestry. We applied strict quality control measures to both genotypes and individuals. For genotypes, variants were retained if the minor allele frequency (MAF) was greater than 0.02, the imputation information score was greater than 0.5, the missing genotype rate was less than 5%, and the Hardy-Weinberg equilibrium P-value was greater than 1 × 10 ⁻ ^7^ [21]. The GRS for each individual is calculated by summing the product of genotypes (0, 1, 2) of risk loci and their corresponding weights, which were from the largest GWAS analysis for RA of European populations [29]. Additionally, we normalized the GRS to a mean of 0 and a standard deviation of 1. We restricted the analysis to approximately 330,000 individuals with successful genotyping, of white British ancestry, and without identified relatives. The best-performing GRS was defined as the score with the strongest association with RA incidence. Finally, genetic risk groups were categorized based on the selected GRS: low genetic risk (bottom quartile), intermediate genetic risk (2nd and 3rd quartiles), and high genetic risk (top quartile). Detailed information on the SNPs used for the GRS is provided in our previous work [30,31].

### Statistical analysis

The basic characteristics of the 379,314 participants were summarized using the mean (standard deviation) for continuous variables and frequencies (percentages) for categorical variables. A Cox regression model was employed to assess the effect of various sleep phenotypes on the risk of RA over time, calculating hazard ratios (HRs) and their corresponding 95% confidence intervals (CIs). Three models were constructed to examine the relationship between sleep behavior and RA risk in this observational study: Model 1, adjusted for genotyping batch (UKB vs. BiLEVE), assessment center, sex, Townsend Deprivation Index (TDI), age, and kinship; Model 2, adjusted for batch, assessment center, sex, TDI, age, kinship, body mass index (BMI), smoking status, and alcohol consumption; Model 3, adjusted for batch, assessment center, sex, TDI, age, kinship, BMI, smoking status, alcohol consumption, diet, physical activity (PA), diastolic blood pressure (DBP), systolic blood pressure (SBP), high-density lipoprotein (HDL) cholesterol, low-density lipoprotein (LDL) cholesterol, triglycerides, and cancer. The TDI is a regionally-based proxy for socioeconomic status. A restricted cubic spline curve and a linearity test were used to assess the non-linear relationship between continuous variables, such as sleep duration and the PSS, and the risk of RA. Incidence rates in different subgroups were evaluated in a prospective study, and Harrell’s c-index was used to evaluate model performance. To assess the potential mediated role of chronic inflammation between sleep and RA, we used an inflammation-based score, the modified Glasgow Prognostic Score (mGPS). Participants with both elevated serum C-reactive protein (CRP) (>1.0 mg/dL) and hypoalbuminemia (<3.5 g/dL) were assigned an mGPS of 2; those with one abnormal value were assigned an mGPS of 1, and those with neither abnormality were assigned an mGPS of 0 [32]. Covariates used in GRS analysis included age, sex, TDI, genotyping batch, and top 10 genetic principal components (PCs). Additionally, we plotted the cumulative incidences of RA based on genetic and sleep risk in this cohort.

In case-control research, propensity score matching (PSM) was used to select participants for the control group, adjusting for genotyping batch, assessment center, sex, TDI, age, race, BMI, smoking status, alcohol consumption, diet, and physical activity. Linear regression was applied to examine the effect of RA onset on sleep duration, while ordinary logistic regression assessed the impact of RA onset on morning wakefulness, chronotype (morning/evening person), daytime napping, insomnia, daytime drowsiness, and PSS. Multivariable logistic regression was then used to evaluate the effect of RA onset on snoring.

Additionally, we plotted the cumulative incidences of RA according to genetic and sleep risk. Statistical significance was defined by a two-tailed P-value < 0.05. To account for multiple comparisons, Bonferroni correction was applied, with a significance threshold of P < 0.006 (0.05/8 sleep behaviors, including PSS). All statistical analyses were conducted using R (version 3.6.1) and PLINK software (version 1.90 b3.38) [33].

## Results

### Baseline characteristics

This study incorporated 379,314 subjects (comprising 171,207 men and 208,107 women), among whom 4,610 were diagnosed with RA. Table 1 shows the baseline characteristics of the study participants in the different PSS groups. Of all participants, 7.09%, 30.40%, 38.45%, 19.75% and 4.30% had a PSS of 0, 1, 2, 3 and 4–5, respectively (S2 Table). As PSS increased, the proportion of regular physical activity and a healthy diet decreased. In contrast, BMI was positively associated with PSS, while the risks of chronic kidney disease, type 2 diabetes, cancer, and RA increased with an increase in PSS.

**Table 1:** Baseline characteristics of the study subjects in the UK Biobank data.

Traits	Level	Poor sleep score		
		Low score	Intermediate score	High score
Number of participants		142,227	220,758	16,329
Age, years (mean (SD))		56.23 (8.24)	56.85 (7.90)	56.59 (7.78)
Male (%)		58235 (42.2)	98157 (46.4)	8243 (53.4)
BMI (mean (SD))		26.49 (4.32)	27.69 (4.78)	29.59 (5.50)
Townsend index (mean (SD))		-1.76 (2.80)	-1.52 (2.96)	-1.02 (3.19)
Tobacco smoking status (%)	Never	81922 (59.4)	108827 (51.4)	6582 (42.6)
	Previous	45827 (33.2)	78903 (37.3)	6298 (40.8)
	Current	10149 (7.4)	23820 (11.3)	2564 (16.6)
Alcohol drinking status (%)	Never	4595 (3.3)	5990 (2.8)	411 (2.7)
	Previous	4246 (3.1)	6785 (3.2)	665 (4.3)
	Current	129015 (93.6)	198682 (93.9)	14359 (93.0)
Regular physical activity (%)		102946 (74.7)	146836 (69.4)	9497 (61.5)
Healthy diet (%)		51896 (37.6)	69215 (32.7)	4209 (27.3)
Chronic kidney disease (%)		3907 (2.8)	7892 (3.7)	834 (5.4)
Type 2 diabetes (%)		4780 (3.5)	11477 (5.4)	1564 (10.1)
Cancer (%)		21620 (15.7)	38251 (18.1)	3199 (20.7)
RA (%)		1387 (1.0)	2903 (1.4)	320 (2.1)
Seropositive RA (%)		170 (0.1)	285 (0.1)	34 (0.2)
Sleep duration (mean (SD))		7.43 (0.62)	7.04 (1.19)	6.78 (1.75)
Sleep duration (%)	7 ~ 8h	131092 (95.1)	120523 (57.0)	683 (4.4)
	7h-	3964 (2.9)	70356 (33.3)	10746 (69.6)
	8h+	2842 (2.1)	20671 (9.8)	4015 (26.0)
The difficulty of getting up (%)	Very easy	57851 (42.0)	59560 (28.2)	2172 (14.1)
	Fairly easy	68858 (49.9)	106307 (50.3)	6873 (44.5)
	Not very easy	9709 (7.0)	36045 (17.0)	4417 (28.6)
	Not at all easy	1480 (1.1)	9638 (4.6)	1982 (12.8)
Chronotype (%)	Definitely morning	50234 (36.4)	45856 (21.7)	558 (3.6)
	Morning more	72403 (52.5)	59555 (28.2)	501 (3.2)
	Evening more	11784 (8.5)	81704 (38.6)	10391 (67.3)
	Definitely evening	3477 (2.5)	24435 (11.6)	3994 (25.9)
Nap during day (%)	Never/rarely	84989 (61.6)	117257 (55.4)	6639 (43.0)
	Sometimes	47601 (34.5)	82989 (39.2)	6841 (44.3)
	Usually	5308 (3.8)	11304 (5.3)	1964 (12.7)
Sleeplessness (%)	Never/rarely	64795 (47.0)	24071 (11.4)	159 (1.0)
	Sometimes	52841 (38.3)	114064 (53.9)	7257 (47.0)
	Usually	20262 (14.7)	73415 (34.7)	8028 (52.0)
Snoring (%)	No	121672 (88.2)	107238 (50.7)	730 (4.7)
	Yes	16226 (11.8)	104312 (49.3)	14714 (95.3)
Daytime dozing (%)	Never/rarely	114064 (82.7)	159619 (75.5)	8500 (55.0)
	Sometimes	23541 (17.1)	46437 (22.0)	3569 (23.1)
	Usually	293 (0.2)	5494 (2.6)	3375 (21.9)

Abbreviation: SD, standard deviation; RA, rheumatoid arthritis; BMI, body mass index.

### Association between sleep behaviors and RA risk

#### Poor sleep conditions and RA risk.

**Fig 2:**
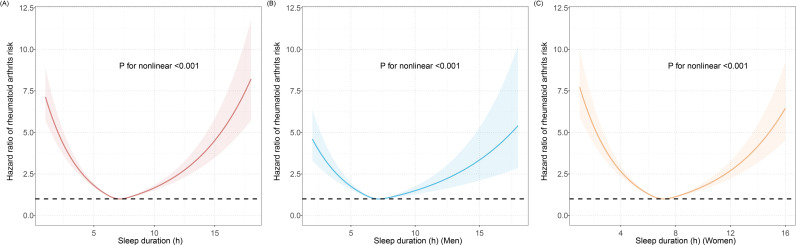
Restricted cubic spline models for the relationship between sleep duration and RA risk. **(A)** All participates; **(B)** In men; **(C)** In women. Note: Associations were adjusted for age, sex, genotyped batch, assessment center, kinship, TDI. Abbreviation: RA, rheumatoid arthritis; TDI, Townsend deprivation index.

In the prospective study, U-shaped relationships between sleep duration and RA risk were observed (*P*_non-linearity_ < 0.001) by using RCS method in the primary analysis (Model 1), with 7h of sleep duration having the lowest risk of RA (Fig 2A). Similar U-shaped associations were observed in sex-stratified groups (*P*_non-linearity_ < 0.001) (Fig 2B and 2C). Participants with short sleep duration (less than 7 hours) and long sleep duration (more than 8 hours) had a higher risk of RA than those who slept 7 to 8 hours per night after multiple test adjustments (HR_sleep duration < 7_ = 1.389, 95%CI = 1.303 ~ 1.480; HR_sleep duration > 8_ = 1.482, 95%CI = 1.352 ~ 1.624) (Table 2). After correcting for more covariates (e.g., BMI, physical activity, and diabetes), the association remained statistically significant in Models 2 and 3. An association between insomnia and RA risk was observed in Model 1. Compared to never/rarely insomnia, the HR estimate for sometimes insomnia was 1.168 (95%CI = 1.077 ~ 1.267) and for frequent insomnia was 1.628 (95%CI = 1.498 ~ 1.769). After correcting for more covariates, the association is barely weakened. In the fully adjusted model (model 3), usually insomnia was associated with incident RA with a 54.4% higher risk (HR: 1.544, 95% CI: 1.404 ~ 1.698). When compared to people who never or seldom encountered daytime dozing, those who usually experienced had a higher risk of developing RA, with the HRs (95% CI) estimated to be 1.683 (1.466 ~ 1.932) in model 1, which is significant after multiple test adjustments (*P* = 1.58 × 10^−13^). Associations were still significant after controlling for more covariates in Models 2 and 3. Besides, we found no evidence of an increased risk of RA due to chronotype and snoring.

**Table 2:** The adjusted hazard ratio for sleep with the risk of RA in the UK Biobank data.

Traits		Model 1		Model 2		Model 3	
		HR (95%CI)	*P*	HR (95%CI)	*P*	HR (95%CI)	*P*
Sleep duration, hours	7 ~ 8	**Ref**		**Ref**		**Ref**	
	<7	1.389 (1.303, 1.480)	**3.16 × 10** ^**-24**^	1.320 (1.238, 1.407)	**2.01 × 10** ^**-17**^	1.332 (1.240, 1.432)	**6.03 × 10** ^**-15**^
	>8	1.482 (1.352, 1.624)	**3.28 × 10** ^**-17**^	1.382 (1.260, 1.515)	**6.56 × 10** ^**-12**^	1.285 (1.154, 1.429)	**4.35 × 10** ^**-06**^
Insomnia	Never/rarely	**Ref**		**Ref**		**Ref**	
	Sometimes	1.168 (1.077, 1.267)	**1.81 × 10** ^**-04**^	1.169 (1.078, 1.269)	**1.77 × 10** ^**-04**^	1.168 (1.065, 1.282)	**9.74 × 10** ^**-04**^
	Usually	1.628 (1.498, 1.769)	**1.65 × 10** ^**-30**^	1.561 (1.435, 1.697)	**2.65 × 10** ^**-25**^	1.544 (1.404, 1.698)	**3.17 × 10** ^**-19**^
Chronotype	Definitely morning	**Ref**		**Ref**		**Ref**	
	Morning more	0.921 (0.858, 0.989)	0.024	0.955 (0.889, 1.026)	0.211	0.957 (0.882, 1.039)	0.294
	Evening more	1.017 (0.944, 1.095)	0.661	1.005 (0.933, 1.084)	0.888	1.010 (0.928, 1.100)	0.811
	Definitely evening	1.087 (0.977, 1.209)	0.127	1.016 (0.912, 1.132)	0.769	1.031 (0.913, 1.163)	0.625
Snoring	No	**Ref**		**Ref**		**Ref**	
	Yes	1.155 (1.089, 1.225)	**1.45 × 10** ^**-06**^	1.038 (0.977, 1.102)	0.229	1.028 (0.960, 1.100)	0.429
Daytime dozing	Never/rarely	**Ref**		**Ref**		**Ref**	
	Sometimes	1.226 (1.149, 1.309)	**9.39 × 10** ^**-10**^	1.185 (1.110, 1.266)	**4.03 × 10** ^**-07**^	1.168 (1.084, 1.259)	**4.70 × 10** ^**-05**^
	Usually	1.683 (1.466, 1.932)	**1.58 × 10** ^**-13**^	1.510 (1.312, 1.737)	**8.75 × 10** ^**-09**^	1.474 (1.257, 1.727)	**1.70 × 10** ^**-06**^
Nap during day	Never/rarely	**Ref**		**Ref**		**Ref**	
	Sometimes	1.200 (1.131, 1.273)	**1.41 × 10** ^**-09**^	1.129 (1.064, 1.199)	**6.71 × 10** ^**-05**^	1.078 (1.007, 1.154)	**0.030**
	Usually	1.487 (1.328, 1.665)	**5.79 × 10** ^**-12**^	1.318 (1.175, 1.478)	**2.35 × 10** ^**-06**^	1.358 (1.197, 1.540)	**1.88 × 10** ^**-06**^
The difficulty of getting up	Very easy	**Ref**		**Ref**		**Ref**	
	Fairly easy	1.092 (1.022, 1.167)	**0.009**	1.095 (1.025, 1.170)	**0.007**	1.067 (0.990, 1.151)	0.088
	Not very easy	1.560 (1.430, 1.702)	**1.02 × 10** ^**-23**^	1.509 (1.383, 1.647)	**2.67 × 10** ^**-20**^	1.484 (1.345, 1.637)	**3.77 × 10** ^**-15**^
	Not at all easy	2.372 (2.106, 2.670)	**3.27 × 10** ^**-46**^	2.131 (1.890, 2.403)	**5.26 × 10** ^**-35**^	1.870 (1.625, 2.152)	**2.62 × 10** ^**-18**^

Note: Model 1: Associations were adjusted for genotype batch, assessment centre, sex, TDI, age, kinship; Model 2: Associations were adjusted for genotype batch, assessment centre, sex, TDI, age, kinship, BMI, smoking status, alcohol; Model 3: Associations were adjusted for genotype batch, assessment centre, sex, TDI, age, kinship, BMI, smoking status, alcohol, diet, PA, DBP, SBP, HDL cholesterol, LDL direct, Cholesterol, Triglycerides, Cancer. Abbreviations: CI, confidence interval; HR, hazard ratio; TDI, Townsend deprivation index; BMI, body mass index; PA, physical activity; DBP, diastolic blood pressure; SBP, systolic blood pressure; HDL, high-density lipoprotein; LDL, low-density lipoprotein; PSS, poor sleep score.

#### Daytime napping and difficulty of getting up and RA risk.

Similarly, naps during day and difficulty in getting up might also contribute to the development of RA. In Model 3, compared to those who never/rarely nap during day, participants who sometimes and usually nap had a 7.8% (HR: 1.078, 95%CI: 1.007 ~ 1.154) and 35.8% (HR: 1.358, 95%CI: 1.197 ~ 1.540) higher risk of developing RA. Interestingly, we also found that difficulty in getting up was also positively associated with the risk of RA, where subjects who got up not at all easy had around 2 times higher risk of RA than those who got up very easily in all models.

#### Sleep behaviors and seropositive RA risk.

We performed a sensitivity analysis to evaluate associations between sleeping conditions and seropositive RA risk. As shown in S3 Table, two sleeping problems (i.e., long sleep duration and insomnia) were also associated with an increased risk of seropositive RA after multiple test adjustments. A long duration of sleep (sleeping ≥ 8h) was associated with an increased risk of seropositive RA. Usually insomnia have a higher risk of RA compared with never/rarely insomnia. Besides, difficulty getting up and daytime napping is also associated with an increased risk of seropositive RA.

### Association between poor sleep score (PSS) and RA risk

**Fig 3:**
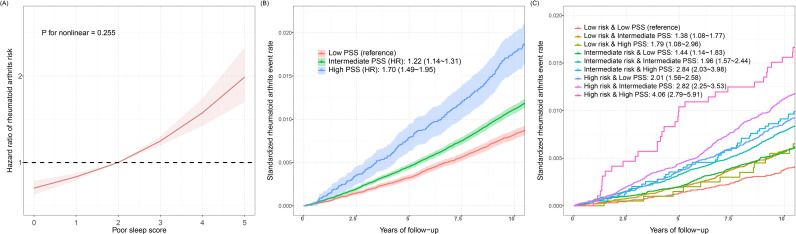
Relationship between poor sleep score (PSS) and RA risk. **(A)** Restricted cubic spline models for the relationship between PSS and RA risk; **(B)** Standardized rates of RA events in low (bottom quartile), intermediate (quartiles 2 to 3), and high (top quartile) PSS groups in the UKB cohort; **(C)** Standardized rates of RA events in participants with different genetic risk score groups and different PSS groups in the UKB cohort. Note: Associations in **A** were adjusted for age, sex, genotyped batch, assessment center, kinship, and TDI. Associations in **B** and **C** were adjusted for genotype batch, assessment centre, sex, TDI, age, kinship, BMI, smoking status, alcohol, diet, PA, DBP, SBP, HDL cholesterol, LDL direct, Cholesterol, Triglycerides, and Cancer. Abbreviations: HR, hazard ratio; TDI, Townsend deprivation index; PA, physical activity; DBP, diastolic blood pressure; SBP, systolic blood pressure; HDL, high-density lipoprotein; LDL, low-density lipoprotein; PSS, poor sleep score.

When a PSS was used to evaluate the combined effects of five sleep behaviors (i.e., sleep duration, insomnia, chronotype, snoring, and daytime dozing) on the risk of RA, we observed linear relationships between PSS and RA risk overall by using RCS method (Fig 3A), as well as sex-stratified groups (S1 Fig). As shown in S4 Table, participants with high PSS have a 70.2% (HR = 1.722, 95% CI: 1.487 ~ 1.949) increased risk of RA compared to those with low scores. Similar results were observed in the different PSS scores (S5 Table) and PSS calculated by cross-validation (PSS_CV_) (S2 Fig). The sensitivity analysis yielded consistent results that people with high PSS_CV_ scores were 60.6% (HR = 1.606, 95%CI: 1.468 ~ 1.757) more likely to develop RA than those with low PSS_CV_ scores. The mediation analyses showed that 2.47% of the effect of PSS on the risk of RA was mediated by mGPS (*P* = 0.002) (S3 Fig). PSS can increase mGPS levels and further increase RA risk (*β*_PSS → mGRS_ = 0.020, *β*_mGRS → RA risk_ = 0.197). We also observed an independent effect of PSS on RA risk after correction for mGPS (*β*_Direct effect_ = 0.154). Linear relationships were also observed in associations between PSS and seropositive RA risk in overall and women (*P*_non-linearity_ > 0.05) but not in men (*P*_non-linearity_ = 0.038) (S3 Fig). As shown in S3 Table, the association between intermediate PSS and seropositive RA no longer existed. Similar results were observed in the different PSS scores (S4 Table) and PSS_CV_ (S3 Table). The cumulative incidence of RA in different PSS groups was shown in Fig 3B.

### The combined effect of sleeping traits and genetic factors on RA risk

After adjustment, we chose the GRS with *P* < 5 × 10^−7^ to represent the genetic component, which had the best performance in terms of the significance of associations (S6 Table). As shown in S7 Table, among all participants, with worsening insomnia, snoring, and daytime dozing, the risk of RA was significantly increased in the moderate GRS group and high GRS group, and participants with high GRS and the worst sleep quality had the highest risk of RA (HR_insomnia_ = 3.652, 95%CI = 2.752 ~ 4.846, HR_snoring_ = 2.306, 95%CI = 1.918 ~ 2.773, HR_daytime dozing_ = 3.805, 95%CI = 2.672 ~ 5.416). For sleep duration and chronotype, with a certain GRS, we also found a U-shaped relationship between sleep duration and RA risk, with the lowest risk observed for 7–8 hours of sleep per night (in the high GRS group, HR_sleep duration7 ~ 8_ = 2.109, 95%CI = 1.789 ~ 2.485, HR_sleep duration < 7_ = 3.158, 95%CI = 2.616 ~ 3.811, HR_sleep duration > 8_ = 2.888, 95%CI = 2.218 ~ 3.761), an evening person were more likely to suffer from RA than morning person (in the high GRS group, HR_definitely morning_ = 2.045, 95%CI = 1.583 ~ 2.642, HR_morning more_ = 2.051, 95%CI = 1.606 ~ 2.619, HR_evening more_ = 2.268, 95%CI = 1.764 ~ 2.916, HR_definitely evening_ = 2.410, 95%CI = 1.741 ~ 3.336). Furthermore, given the same sleep conditions, we found that the high GRS group had a higher risk of RA than the medium GRS group, whereas no such results were observed in the low GRS group (except for sleep duration). In the sex-stratified analysis, the combined effect of sleep factors and GRS was greater in women than in men.

**Fig 4:**
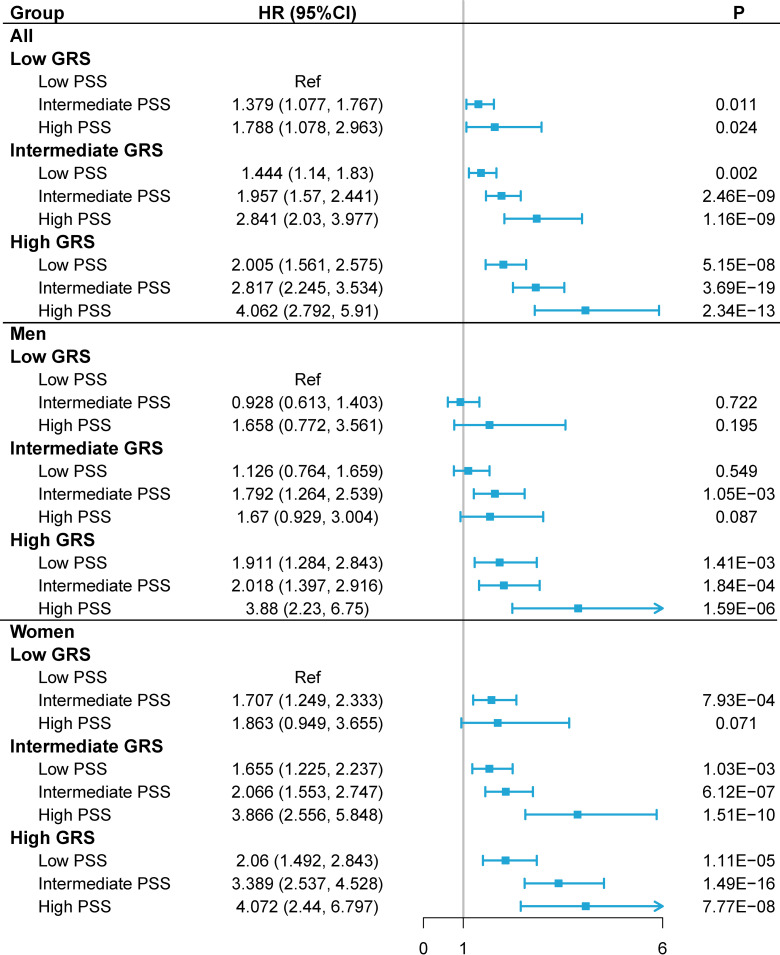
RA risks in the subgroups stratified by genetic risk and poor sleep score (PSS) (versus participants with the lowest PSS in the lowest genetic risk group) in the UKB cohort. Note: Associations were adjusted for age, sex, TDI, genotyping batch, and top 10 genetic PCs. Abbreviations: PSS, poor sleep score; CI, confidence interval; HR, hazard ratio; PC, principal components; GRS, genetic risk score.

We next assessed the combined association of PSS and GRS on the risk of developing RA, detailed results were presented in Fig 4. According to our findings, the combined effect of GRS and PSS enhanced the chance of RA incident. We also discovered that subjects with high PSS and high GRS had the highest risk of developing RA (HR = 4.062, 95%CI: 2.792 ~ 5.910). Results stratified by gender showed that women were more likely than men to be affected by the combined effects of PSS and GRS. In the high PSS and high GRS group, the HRs (95% CI) were estimated to be 3.880 (2.230 ~ 6.750) for men, and 4.072 (2.440 ~ 6.797) for women. Similar results were also observed with the PSS calculated by using cross-validation method (S4 Fig). Fig 3C shows the cumulative incidence of RA events in participants with different GRS groups and different PSS groups. C-statistic was calculated to be 0.657 in model without GRS and 0.672 in model with GRS (2.2% improvement).

### Effect of RA onset on sleep condition

As shown in S8 Table, in the case-control study, there were statistically significant associations between RA onset and poor sleep conditions (i.e., PSS) (*β* = 0.191, 95%CI = 0.095 ~ 0.288). Besides, ordinal regression analysis indicated that RA onset could increase the risk of difficulty getting up (OR = 1.293, 95%CI = 1.156 ~ 1.446,). However, no significant associations were observed between RA onset and other sleep behaviors, including insomnia, sleep duration, and daytime drowsiness, in later life.

### Genetic correlations between RA and sleep phenotypes

In the analysis of genetic correlations using summary data from GWAS, we observed positive genetic associations between RA and insomnia (rg = 0.13; *P* = 0.0002) and chronotype (rg = 0.1; *P* = 0.00008), as well as negative genetic associations with sleep duration. Similar results were observed in seropositive RA (S5 Fig provides detailed information).

## Discussion

This study, utilizing large-scale data from the UK Biobank, this study systematically explored the relationship between seven sleep behaviors and RA risk. The prospective study indicated that abnormal sleep duration, insomnia, and daytime dozing could increase the risk of RA. A U-shaped relationship between sleep duration and RA risk, with the lowest risk of RA when the daily sleep duration was 7–8 hours. Additionally, a PSS was developed to evaluate the combined effects of five poor sleep behaviors, revealing that higher PSS values were linked to an increased RA risk. In particular, individuals in the highest PSS group were found to have a 70.2% greater likelihood of developing RA. We also conducted a mediation analysis, which revealed that the mGPS acted as a mediator between sleep behaviors and RA risk. Furthermore, we observed the combined effects of genetic factors and PSS on RA risk, with sex-stratified analysis showing a stronger effect in women than in men. The case-control study further confirmed that RA risk is associated with long sleep duration, insomnia, and difficulty waking up in the morning. Lastly, significant genetic correlations were identified between sleep phenotypes and various types of RA.

Our findings provide robust evidence supporting the association between poor sleep and an elevated risk of RA, particularly with abnormal sleep duration, insomnia, and daytime drowsiness. A study conducted in China similarly found that poor sleep quality and short sleep duration increase RA risk [34], consistent with our results. However, we identified a U-shaped relationship between sleep duration and RA risk, with the lowest risk found in individuals who slept 7–8 hours per day. This pattern is consistent with studies on other diseases and sleep duration. For instance, research by Abbas Smiley et al. showed that women with extreme sleep durations had an increased likelihood of developing metabolic syndrome, with the lowest severity scores observed among those sleeping 7 hours per night [12]. Similarly, a meta-analysis reported a U-shaped relationship between sleep duration and osteoporosis risk, with the lowest risk for 8 hours of sleep per night [13]. A cohort study of 65,754 patients in Taiwan found a link between sleep disorders and a higher risk of RA [16], and a study by a conclusion supported by Taiwan’s National Health Insurance Scheme [17]. A UK cohort study revealed that faster cognitive decline in individuals who slept fewer than 4 hours or more than 10 hours per night, compared to those who slept 7 hours [14]. Jiawei Yin *et al.* also demonstrated that both insufficient and excessive sleep increase all-cause mortality and cardiovascular events, with 7 hours of sleep per night associated with the lowest risk [15]. Notably, we also observed significant associations between poor sleep patterns and RA risk in seropositive RA, especially long sleep duration. Furthermore, a cohort study from the Taiwan National Health Insurance Research Database suggested that obstructive sleep apnea, a sleep disorder linked to snoring, may increase RA risk, although treating OSA could mitigate this risk [35]. However, we did not find a significant association between snoring and RA risk, which may be due to the self-reported nature of snoring data and the lack of detailed information such as snoring frequency and duration.

The PSS constructed in our study is essential for assessing individuals’ sleep patterns. Sleep behaviors are interrelated and can influence one another, with the body holistically regulating sleep. Changes in one sleep factor often led to compensatory changes in other [36,37]. For example, insomnia could result in shorter sleep duration and increased daytime drowsiness. The PSS developed here provides a comprehensive measure of sleep behavior, capturing the combined impact of five poor sleep factors on RA risk. This score offers a useful framework for assessing overall sleep quality, allowing for more accurate identification of high-risk individuals. Moreover, the simplicity of the scoring algorithm enhances the clarity and applicability of epidemiological findings, increasing their information value [36].Recent studies investigating the relationship between RA and sleep, such as that by Liu et al. [38], have provided valuable insights. Our study contributes to this field by introducing several innovative approaches. First, we included a sensitivity analysis focused on seropositive RA, enriching our understanding of the impact on different RA subtypes. Second, we performed a mediation analysis, which demonstrated that the mGPS acts as a mediator between sleep behaviors and RA risk. Third, we conducted a PSM case-control study to minimize confounding, thus strengthening the reliability of our results. Finally, we employed LDSC to explore genetic correlations between sleep behaviors and RA, uncovering potential shared genetic mechanisms. These methodological innovations help further elucidate the complex interactions between sleep patterns, genetic predisposition, and RA.

Several mechanisms may link sleep disturbances to RA risk, including chronic inflammation. We found that the mGPS, an inflammation-based score, mediated the relationship between sleep and RA risk, although the effect was modest. Sleep and immune system disorders are linked in both directions. Sleep disturbances and immune system disorders are bidirectional: a disrupted immune system can impair sleep, while poor sleep, in turn, can affect immune function [39]. Previous studies have suggested that insomnia and other non-apnea sleep disorders may increase the risk of autoimmune diseases, including RA, systemic lupus erythematosus, and ankylosing spondylitis [17]. Chronic low-grade inflammation associated with poor sleep is thought to contribute to RA risk [40]. Immunological studies indicate that sleep deprivation can elevate the number of circulating T cells and B cells, while disrupting the rhythm of CD4+ regulatory T cells (Tregs) [41,42]. Activated T cells can stimulate osteoclasts, leading to joint destruction. Additionally, the interaction between T cells and B cells can perpetuate the degradation of the synovial membrane [43]. Furthermore, poor sleep can upregulate inflammatory markers such as tumor necrosis factor-alpha (TNF-α), interleukin (IL)-1, IL-6, IL-8, and IL-17 [44,45]. The finding that women generally exhibit higher levels of inflammatory markers aligns with our observation that women with poor sleep quality may be at greater risk for RA [46]. Dysregulation of IL-6 and TNF-α is known to disrupt the balance between cartilage and bone matrix degradation in joints [47]. Notably, an animal study confirmed that IL-17 levels remained elevated in sleep-deprived rats even after 7 days of recovery [48]. IL-17 not only triggers an inflammatory response but also enhances RANKL sensitivity by modulating RANK expression, which increases osteoclast activity and bone resorption [49].

### Strength and limitations

This study has several strengths. First, we assessed the relationship between sleep and various types of RA by integrating prospective cohort and PSM case-control studies. Second, we developed a sleep score to evaluate the association between overall sleep patterns and RA risk, incorporating multiple sleep behaviors. Third, we explored the combined effects of sleep patterns and genetic risk on RA. Additionally, we identified significant genetic correlations between sleep phenotypes and different RA subtypes. However, our study also has limitations. First, it was conducted among European Caucasians, limiting the generalizability of the findings to other racial groups. Second, sleep patterns were assessed only at baseline, and the use of a single baseline measure to create the sleep score does not account for changes in sleep behaviors over time. Future studies should incorporate multiple time points to better understand how changes in sleep behavior affect RA risk. Third, while we adjusted for known confounders, there may still be unmeasured or unknown confounding factors. Finally, the sleep data were self-reported, introducing potential measurement errors.

## Supporting information

S1 Fig Restricted cubic spline models for the relationship between PSS and RA risk in men (A) and women (B). Note: Associations were adjusted for age, sex, genotyped batch, assessment center, kinship, and TDI. Abbreviation: TDI, Townsend deprivation index.
(TIF)

S2 Fig The contribution of mGPS for the association between PSS and RA risk. Note: * *p* < 0.05, **p < 0.01, ***p < 0.001. Associations were adjusted for age, sex, genotyped batch, assessment center, kinship, and TDI. Abbreviation: RA, rheumatoid arthritis; PSS, poor sleep score; mGPS, modified Glasgow Prognostic Score; TDI, Townsend deprivation index.
(TIF)

S3 Fig Restricted cubic spline models for the relationship between PSS and seropositive RA risk. (A) All participants; (B) In men; (C) In women. Note: Associations were adjusted for age, sex, genotyped batch, assessment center, kinship, and TDI. Abbreviation: TDI, Townsend deprivation index.
(TIF)

S4 Fig RA risks in the subgroups stratified by genetic risk and PSSCV (versus participants with lowest PSS in the lowest genetic risk group) in the UKB cohort. Note: Associations were adjusted for age, sex, TDI, genotyping batch, and top 10 genetic PCs. Abbreviations: PSS, poor sleep score; CI, confidence interval; HR, hazard ratio; PC, principal components; GRS, genetic risk score; CV, cross-validation.
(TIF)

S5 Fig Genetic correlation of sleep phenotypes with different types of RA (*P* < 0.05). Abbreviation: RA; rheumatoid arthritis.
(TIF)

S1 Table Description and definition of sleeping behaviors in UK Biobank data. (PDF)

S2 Table Baseline characteristics of the study subjects stratified by different PSS scores in the UK Biobank data. Abbreviation: SD, standard deviation; RA, rheumatoid arthritis; BMI, body mass index; PSS, poor sleep score.
(PDF)

S3 Table The adjusted hazard ratio for sleep behaviors on the risk of seropositive RA in the UK Biobank data. Note: Model 1: Associations were adjusted for genotype batch, assessment centre, sex, TDI, age, kinship; Model 2: Associations were adjusted for genotype batch, assessment centre, sex, TDI, age, kinship, BMI, smoking status, alcohol; Model 3: Associations were adjusted for genotype batch, assessment centre, sex, TDI, age, kinship, BMI, smoking status, alcohol, diet, PA, DBP, SBP, HDL cholesterol, LDL direct, Cholesterol, Triglycerides, Cancer. Abbreviations: CI, confidence interval; HR, hazard ratio; TDI, Townsend deprivation index; BMI, body mass index; PA, physical activity; DBP, diastolic blood pressure; SBP, systolic blood pressure; HDL, high-density lipoprotein; LDL, low-density lipoprotein; PSS, poor sleep score; CV, cross-validation.
(PDF)

S4 Table The adjusted hazard ratio for RA and seropositive RA risk stratified by three PSS categories in the UK Biobank data . Note: Model 1: Associations were adjusted for genotype batch, assessment centre, sex, TDI, age, kinship; Model 2: Associations were adjusted for genotype batch, assessment centre, sex, TDI, age, kinship, BMI, smoking status, alcohol; Model 3: Associations were adjusted for genotype batch, assessment centre, sex, TDI, age, kinship, BMI, smoking status, alcohol, diet, PA, DBP, SBP, HDL cholesterol, LDL direct, Cholesterol, Triglycerides, Cancer. Abbreviations: CI, confidence interval; HR, hazard ratio; TDI, Townsend deprivation index; BMI, body mass index; PA, physical activity; DBP, diastolic blood pressure; SBP, systolic blood pressure; HDL, high-density lipoprotein; LDL, low-density lipoprotein; PSS, poor sleep score; CV, cross-validation.
(PDF)

S5 Table The adjusted hazard ratio for RA and seropositive RA risk stratified by five PSS categories in the UK Biobank data. Note: Model 1: Associations were adjusted for genotype batch, assessment centre, sex, TDI, age, kinship; Model 2: Associations were adjusted for genotype batch, assessment centre, sex, TDI, age, kinship, BMI, smoking status, alcohol; Model 3: Associations were adjusted for genotype batch, assessment centre, sex, TDI, age, kinship, BMI, smoking status, alcohol, diet, PA, DBP, SBP, HDL cholesterol, LDL direct, Cholesterol, Triglycerides, Cancer. Abbreviations: CI, confidence interval; HR, hazard ratio; TDI, Townsend deprivation index; BMI, body mass index; PA, physical activity; DBP, diastolic blood pressure; SBP, systolic blood pressure; HDL, high-density lipoprotein; LDL, low-density lipoprotein; PSS, poor sleep score.
(PDF)

S6 Table The adjusted hazard ratio for the effect of GRSs in different cutoffs on RA risk in both training set and test set. Note: Associations were adjusted for age, sex, TDI, genotyping chip (UKB vs BiLEVE), and top 10 genetic PCs. Abbreviations: CI, confidence interval; HR, hazard ratio; GRS, genetic risk score; TDI, Townsend deprivation index; PC, principal components.
(PDF)

S7 Table RA risks in the subgroups stratified by genetic risk and different sleep behaviors (versus participants with healthy sleep behaviors in the lowest genetic risk group) in the UK Biobank. Note: Associations were adjusted for age, sex, TDI, genotyping batch, and top 10 genetic PCs. Abbreviations: CI, confidence interval; HR, hazard ratio; PC, principal components; GRS, genetic risk score.
(PDF)

S8 Table The effect of RA onset on sleep condition in the UK Biobank data. Note: # represents the utilization of linear regression due to the continuous Abbreviations: CI, confidence interval; PSS, poor sleep score; CV, cross-validation.
(PDF)
