# Supplementary figures and images for: Poor sleep and high rheumatoid arthritis risk: Evidence from large UK Biobank cohort
Source: PLoS One. 2025 Apr 23;20(4):e0318728. doi: 10.1371/journal.pone.0318728 (PMC12017501; doi:10.1371/journal.pone.0318728)

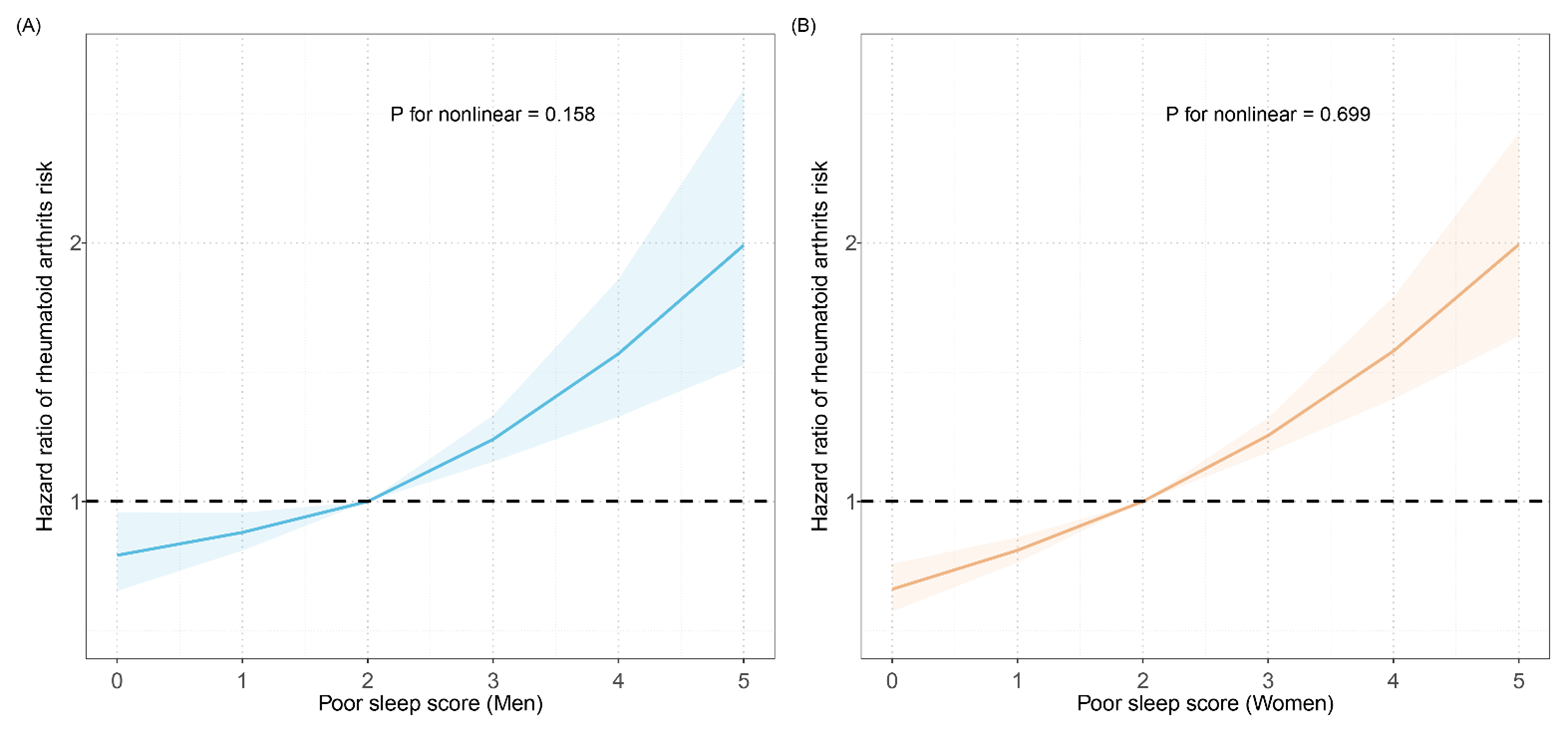

Supplement: S1 Fig — Note: Associations were adjusted for age, sex, genotyped batch, assessment center, kinship, and TDI. Abbreviation: TDI, Townsend deprivation index. (TIF) [file pone.0318728.s001.tif]

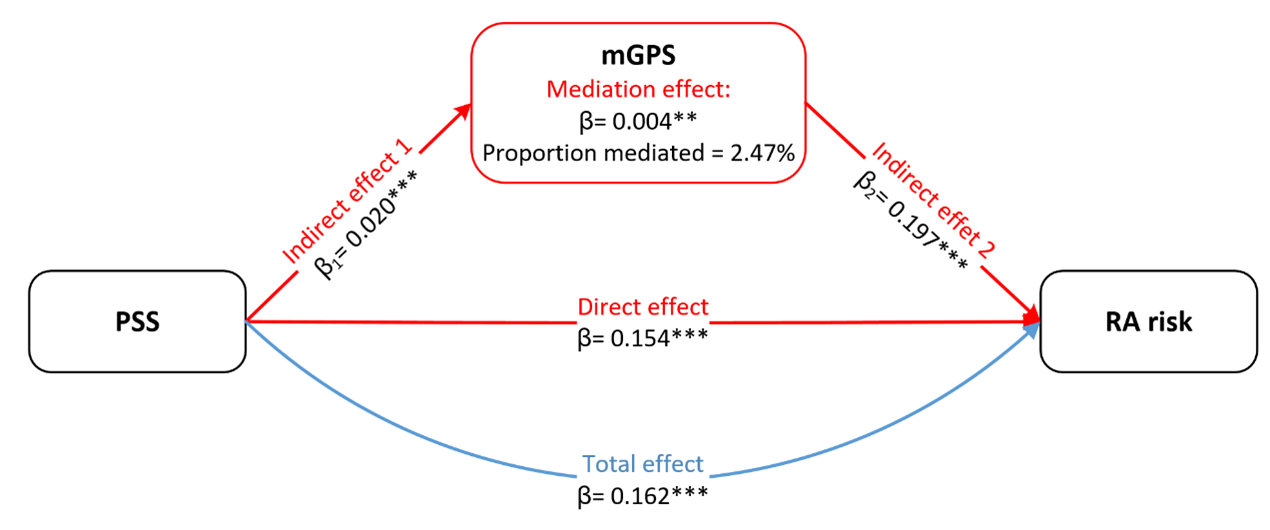

Supplement: S2 Fig — Note: * p < 0.05, **p < 0.01, ***p < 0.001. Associations were adjusted for age, sex, genotyped batch, assessment center, kinship, and TDI. Abbreviation: RA, rheumatoid arthritis; PSS, poor sleep score; mGPS, modified Glasgow Prognostic Score; TDI, Townsend deprivation index. (TIF) [file pone.0318728.s002.tif]

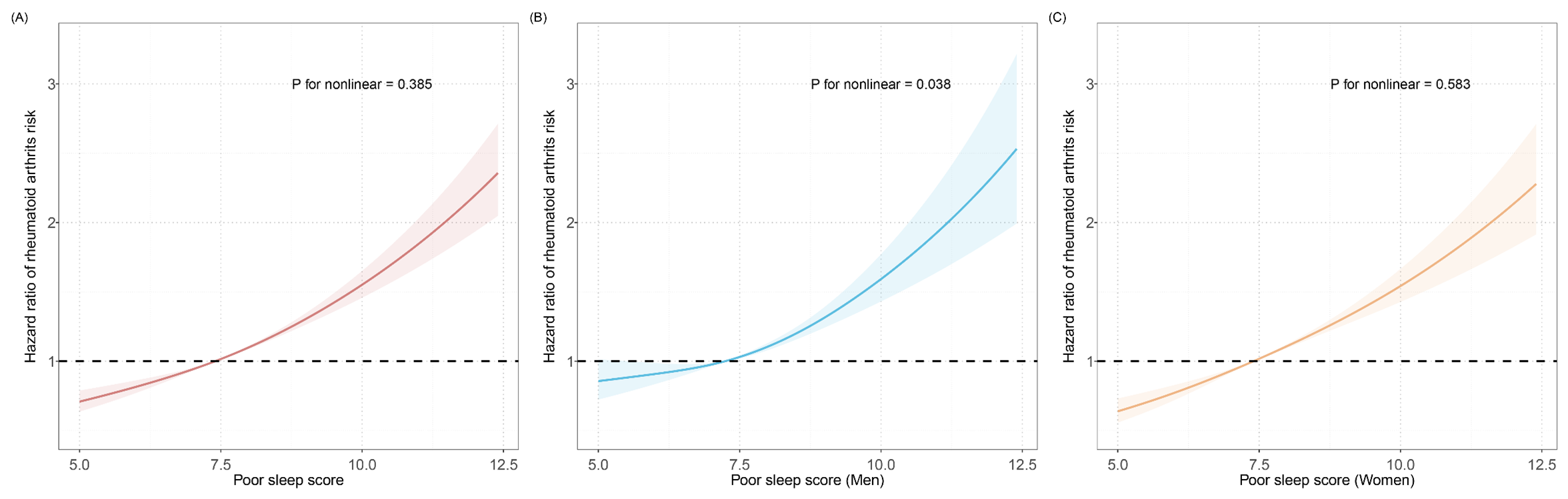

Supplement: S3 Fig — (A) All participants; (B) In men; (C) In women. Note: Associations were adjusted for age, sex, genotyped batch, assessment center, kinship, and TDI. Abbreviation: TDI, Townsend deprivation index. (TIF) [file pone.0318728.s003.tif]

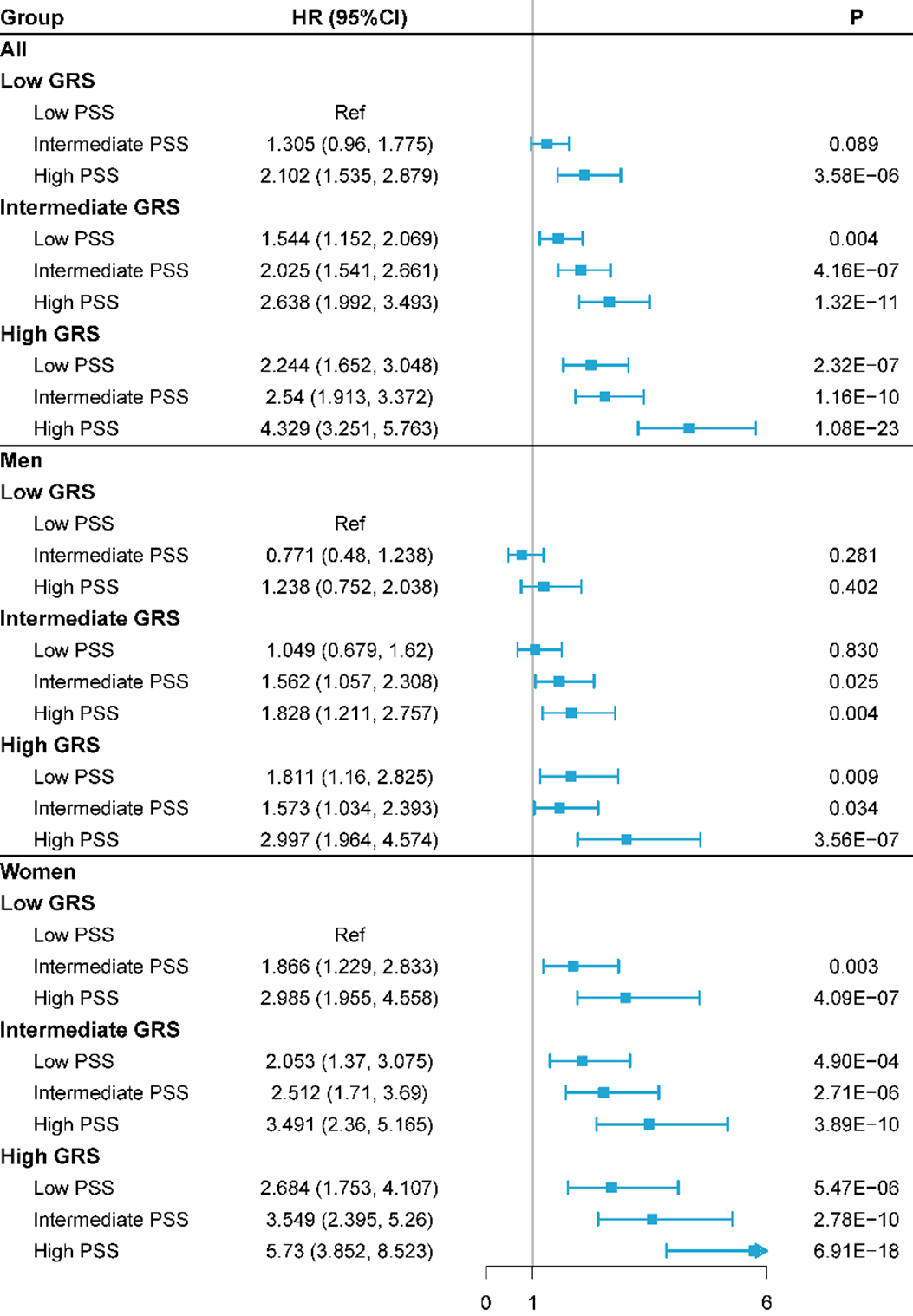

Supplement: S4 Fig — Note: Associations were adjusted for age, sex, TDI, genotyping batch, and top 10 genetic PCs. Abbreviations: PSS, poor sleep score; CI, confidence interval; HR, hazard ratio; PC, principal components; GRS, genetic risk score; CV, cross-validation. (TIF) [file pone.0318728.s004.tif]

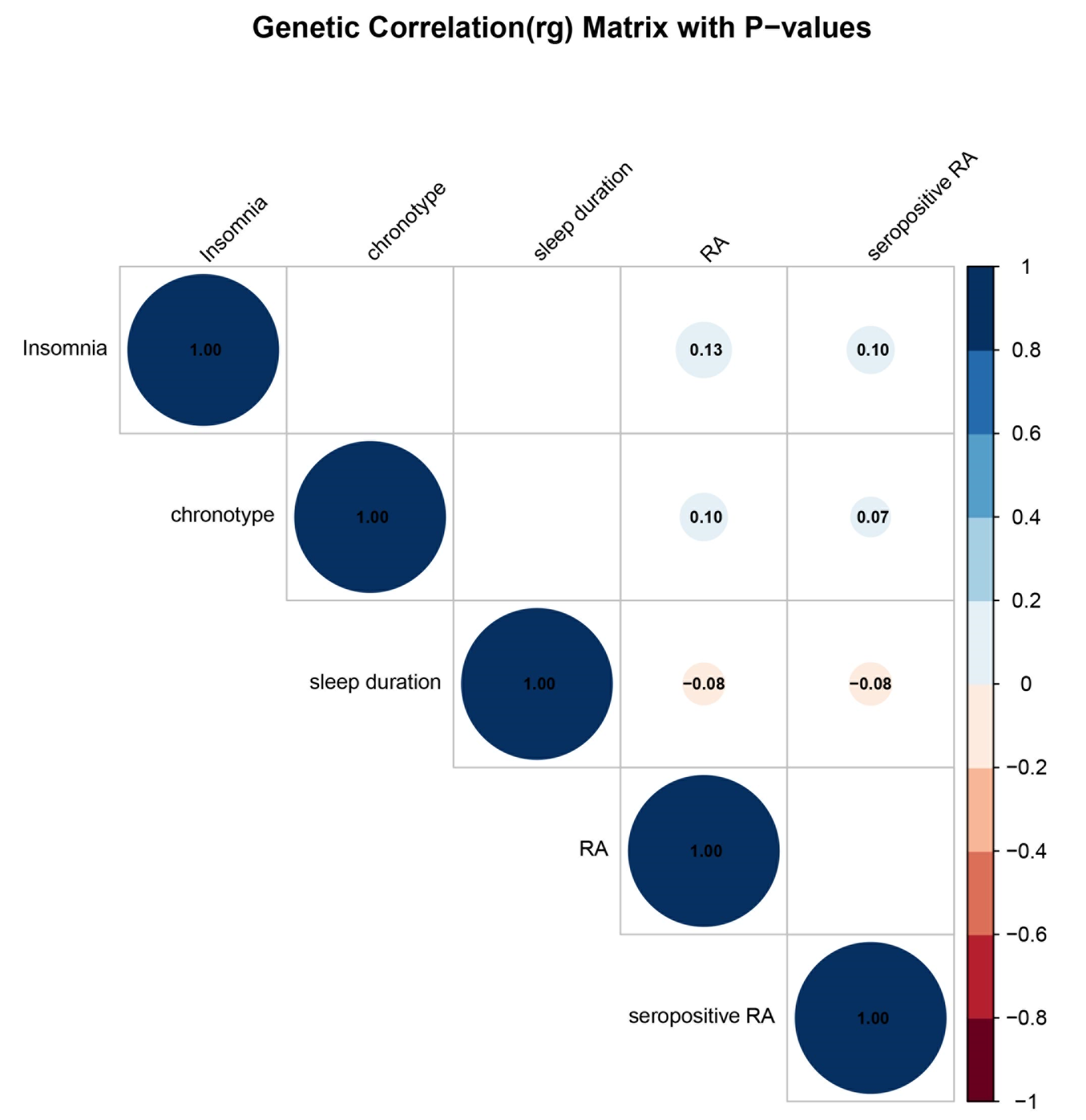

Supplement: S5 Fig — Abbreviation: RA; rheumatoid arthritis. (TIF) [file pone.0318728.s005.tif]
