# Supplementary material for: Poor sleep and high rheumatoid arthritis risk: Evidence from large UK Biobank cohort
Source: PLoS One. 2025 Apr 23;20(4):e0318728. doi: 10.1371/journal.pone.0318728 (PMC12017501; doi:10.1371/journal.pone.0318728)
Supplement: S1 Table — (PDF) [file pone.0318728.s006.pdf]

Sleeping factor	Source	Definition	UK Biobank field codes
Sleep duration	About how many hours of sleep do you get in every 24 hours?	7~8h<br><7h<br>>8h	1160
Getting up in morning	On an average day, how easy do you find getting up in the morning?	1: Not at all easy<br>2: Not very easy<br>3: Fairly easy<br>4: Very easy	1170
Morning/evening person	Do you consider yourself to be?	1: Definitely morning<br>2: Morning more<br>3: Evening more<br>4: Definitely evening	1180
Nap during day	Do you have a nap during the day?	1: Never/rarely<br>2: Sometimes<br>3: Usually	1190
Sleeplessness/insomnia	Do you have trouble falling asleep at night or do you wake up in the middle of the night?	1: Never/rarely<br>2: Sometimes<br>3: Usually	1200
Snoring	Does your partner or a close relative or friend complain about your snoring?	1: Yes<br>2: No	1210
Daytime dozing/sleeping	How likely are you to doze off or fall asleep during the daytime when you don't mean to? (e.g. when working, reading or driving)	0: Never/rarely<br>1: Sometimes<br>2: Usually	1220
