# Supplementary material for: Poor sleep and high rheumatoid arthritis risk: Evidence from large UK Biobank cohort
Source: PLoS One. 2025 Apr 23;20(4):e0318728. doi: 10.1371/journal.pone.0318728 (PMC12017501; doi:10.1371/journal.pone.0318728)
Supplement: S2 Table — Abbreviation: SD, standard deviation; RA, rheumatoid arthritis; BMI, body mass index; PSS, poor sleep score. (PDF) [file pone.0318728.s007.pdf]

Traits	level	PSS				
		0	1	2	3	4-5
Number of participants		26,899	115,328	145,841	74,917	16,329
Age, years (mean (SD))		55.14 (8.55)	56.58 (8.15)	56.95 (7.94)	56.85 (7.84)	56.65 (7.80)
Sex (%)	Female	14639 (54.4)	67440 (58.5)	80607 (55.3)	37674 (50.3)	7655 (46.9)
	Male	12264 (45.6)	47905 (41.5)	65185 (44.7)	37220 (49.7)	8672 (53.1)
BMI (mean (SD))		26.11 (4.18)	26.63 (4.38)	27.40 (4.67)	28.39 (5.01)	29.65 (5.53)
Townsend index (mean (SD))		-1.75 (2.83)	-1.72 (2.82)	-1.55 (2.94)	-1.32 (3.07)	-0.96 (3.22)
Tobacco smoking status (%)	Never	16515 (63.0)	65407 (58.6)	74708 (53.3)	34119 (47.8)	6582 (42.6)
	Previous	7896 (30.1)	37931 (34.0)	51474 (36.7)	27429 (38.4)	6298 (40.8)
	Current	1798 (6.9)	8351 (7.5)	14017 (10.0)	9803 (13.7)	2564 (16.6)
Alcohol drinking status (%)	Never	961 (3.7)	3634 (3.3)	4084 (2.9)	1906 (2.7)	411 (2.7)
	Previous	836 (3.2)	3410 (3.1)	4375 (3.1)	2410 (3.4)	665 (4.3)
	Current	24404 (93.1)	104611 (93.7)	131680 (93.9)	67002 (93.9)	14359 (93.0)
Regular physical activity (%)		20318 (77.5)	82628 (74.0)	99098 (70.7)	47738 (66.9)	9497 (61.5)
Healthy diet (%)		10314 (39.4)	41582 (37.2)	47654 (34.0)	21561 (30.2)	4209 (27.3)
Chronic kidney disease (%)		607 (2.3)	3536 (3.1)	5262 (3.6)	3255 (4.3)	916 (5.6)
Type 2 diabetes (%)		763 (2.8)	4334 (3.8)	7190 (4.9)	5204 (6.9)	1692 (10.4)
Cancer (%)		3819 (14.2)	18699 (16.2)	25964 (17.8)	14505 (19.4)	3432 (21.0)
RA (%)		232 (0.9)	1216 (1.1)	1942 (1.3)	1163 (1.6)	336 (2.1)
Seropositive RA (%)		25 (0.1)	145 (0.1)	187 (0.1)	98 (0.1)	34 (0.2)
Sleep duration (mean (SD))		7.50 (0.50)	7.42 (0.64)	7.12 (1.08)	6.89 (1.40)	6.79 (1.77)
Sleep duration (%)	7~8h	26903 (100.0)	108255 (93.9)	97294 (66.7)	27643 (36.9)	720 (4.4)
	7h-	0 (0.0)	4116 (3.6)	37877 (26.0)	36048 (48.1)	11306 (69.2)
	8h+	0 (0.0)	2974 (2.6)	10621 (7.3)	11203 (15.0)	4301 (26.3)

The difficulty of getting up (%)	Very easy	13458 (50.0)	46412 (40.2)	45832 (31.4)	16557 (22.1)	2290 (14.0)
	Fairly easy	12291 (45.7)	58557 (50.8)	73907 (50.7)	36724 (49.0)	7221 (44.2)
	Not very easy	1053 (3.9)	8917 (7.7)	21094 (14.5)	16393 (21.9)	4677 (28.6)
	Not at all easy	101 (0.4)	1459 (1.3)	4959 (3.4)	5220 (7.0)	2139 (13.1)
Chronotype (%)	Definitely morning	11590 (43.1)	40644 (35.2)	37712 (25.9)	10735 (14.3)	596 (3.7)
	Morning more	15313 (56.9)	59044 (51.2)	49469 (33.9)	12501 (16.7)	531 (3.3)
	Evening more	0 (0.0)	12086 (10.5)	45736 (31.4)	39219 (52.4)	10973 (67.2)
	Definitely evening	0 (0.0)	3571 (3.1)	12875 (8.8)	12439 (16.6)	4227 (25.9)
Nap during day (%)	Never/rarely	17882 (66.5)	69365 (60.1)	82676 (56.7)	38804 (51.8)	6925 (42.4)
	Sometimes	8103 (30.1)	41365 (35.9)	56128 (38.5)	31111 (41.5)	7273 (44.5)
	Usually	918 (3.4)	4615 (4.0)	6988 (4.8)	4979 (6.6)	2129 (13.0)
Sleeplessness (%)	Never/rarely	26903 (100.0)	39832 (34.5)	20959 (14.4)	3991 (5.3)	166 (1.0)
	Sometimes	0 (0.0)	54589 (47.3)	79027 (54.2)	39817 (53.2)	7639 (46.8)
	Usually	0 (0.0)	20924 (18.1)	45806 (31.4)	31086 (41.5)	8522 (52.2)
Snoring (%)	No	26903 (100.0)	98562 (85.4)	88219 (60.5)	23772 (31.7)	788 (4.8)
	Yes	0 (0.0)	16783 (14.6)	57573 (39.5)	51122 (68.3)	15539 (95.2)
Daytime dozing (%)	Never/rarely	23326 (86.7)	94004 (81.5)	112751 (77.3)	52975 (70.7)	8906 (54.5)
	Sometimes	3577 (13.3)	21039 (18.2)	30972 (21.2)	18171 (24.3)	3800 (23.3)
	Usually	0 (0.0)	302 (0.3)	2069 (1.4)	3748 (5.0)	3621 (22.2)
