# Supplementary material for: Poor sleep and high rheumatoid arthritis risk: Evidence from large UK Biobank cohort
Source: PLoS One. 2025 Apr 23;20(4):e0318728. doi: 10.1371/journal.pone.0318728 (PMC12017501; doi:10.1371/journal.pone.0318728)
Supplement: S3 Table — Note: Model 1: Associations were adjusted for genotype batch, assessment centre, sex, TDI, age, kinship; Model 2: Associations were adjusted for genotype batch, assessment centre, sex, TDI, age, kinship, BMI, smoking status, alcohol; Model 3: Associations were adjusted for genotype batch, assessment centre, sex, TDI, age, kinship, BMI, smoking status, alcohol, diet, PA, DBP, SBP, HDL cholesterol, LDL direct, Cholesterol, Triglycerides, Cancer. Abbreviations: CI, confidence interval; HR, hazard ratio; TDI, Townsend deprivation index; BMI, body mass index; PA, physical activity; DBP, diastolic blood pressure; SBP, systolic blood pressure; HDL, high-density lipoprotein; LDL, low-density lipoprotein; PSS, poor sleep score; CV, cross-validation. (PDF) [file pone.0318728.s008.pdf]

Sleep behaviors	Levels	**Model 1**		**Model 2**		**Model 3**	
		**HR (95%CI)**	***P***	**HR (95%CI)**	***P***	**HR (95%CI)**	***P***
Sleep duration	7~8h	Ref		Ref		Ref	
	7h-	1.163 (0.994, 1.361)	0.059	1.110 (0.947, 1.300)	0.199	1.055 (0.879, 1.265)	0.567
	8h+	1.768 (1.450, 2.157)	1.86E -08	1.657 (1.355, 2.027)	8.85E-07	1.478 (1.168, 1.872)	1.17E-03
Getting up	Very easy	Ref		Ref		Ref	
	Fairly easy	1.281 (1.089, 1.507)	0.003	1.293 (1.098, 1.523)	0.002	1.310 (1.086, 1.580)	0.005
	Not very easy	2.032 (1.660, 2.487)	6.03E-12	1.958 (1.596, 2.401)	1.11E-10	2.052 (1.629, 2.585)	1.02E-09
	Not at all easy	2.805 (2.122, 3.708)	4.36E-13	2.589 (1.953, 3.433)	3.85E-11	2.224 (1.586, 3.118)	3.60E-06
chronotype	Definitely morning	Ref		Ref		Ref	
	Morning more	0.960 (0.810, 1.138)	0.640	0.998 (0.841, 1.185)	0.983	1.045 (0.858, 1.273)	0.662
	Evening more	1.030 (0.862, 1.231)	0.744	1.018 (0.850, 1.219)	0.845	1.086 (0.884, 1.335)	0.432
	Definitely evening	1.353 (1.067, 1.715)	0.012	1.259 (0.990, 1.602)	0.061	1.208 (0.911, 1.601)	0.190
Nap during day	Never/rarely	Ref		Ref		Ref	
	Sometimes	1.236 (1.074, 1.423)	0.003	1.196 (1.037, 1.378)	0.014	1.110 (0.944, 1.306)	0.207
	Usually	1.983 (1.552, 2.533)	4.35E-08	1.769 (1.375, 2.275)	8.91E-06	1.644 (1.232, 2.195)	7.40E-04
Insomnia	Never/rarely	Ref		Ref		Ref	
	Sometimes	1.150 (0.951, 1.391)	0.151	1.148 (0.948, 1.390)	0.158	1.266 (1.012, 1.583)	0.039
	Usually	1.533 (1.260, 1.865)	1.94E-05	1.474 (1.209, 1.796)	1.21×10-04	1.560 (1.237, 1.967)	1.68E-04
Snoring	No	Ref		Ref		Ref	
	Yes	1.039 (0.902, 1.197)	0.594	0.993 (0.860, 1.148)	0.929	0.932 (0.789, 1.101)	0.408
Daytime dozing	Never/rarely	Ref		Ref		Ref	
	Sometimes	1.204 (1.032, 1.405)	0.018	1.187 (1.016, 1.386)	0.031	1.191 (0.997, 1.422)	0.054
	Usually	1.177 (0.800, 1.732)	0.407	1.131 (0.768, 1.666)	0.532	0.902 (0.555, 1.467)	0.678
