# Supplementary material for: Poor sleep and high rheumatoid arthritis risk: Evidence from large UK Biobank cohort
Source: PLoS One. 2025 Apr 23;20(4):e0318728. doi: 10.1371/journal.pone.0318728 (PMC12017501; doi:10.1371/journal.pone.0318728)
Supplement: S4 Table — . Note: Model 1: Associations were adjusted for genotype batch, assessment centre, sex, TDI, age, kinship; Model 2: Associations were adjusted for genotype batch, assessment centre, sex, TDI, age, kinship, BMI, smoking status, alcohol; Model 3: Associations were adjusted for genotype batch, assessment centre, sex, TDI, age, kinship, BMI, smoking status, alcohol, diet, PA, DBP, SBP, HDL cholesterol, LDL direct, Cholesterol, Triglycerides, Cancer. Abbreviations: CI, confidence interval; HR, hazard ratio; TDI, Townsend deprivation index; BMI, body mass index; PA, physical activity; DBP, diastolic blood pressure; SBP, systolic blood pressure; HDL, high-density lipoprotein; LDL, low-density lipoprotein; PSS, poor sleep score; CV, cross-validation. (PDF) [file pone.0318728.s009.pdf]

Group		Model 1		Model 2		Model 3	
		**HR (95%CI)**	***P***	**HR (95%CI)**	***P***	**HR (95%CI)**	***P***
**RA**							
PSS (linear)		1.226 (1.191, 1.261)	1.77E-43	1.161 (1.127, 1.196)	2.58E-23	1.156 (1.118, 1.195)	1.83E-17
PSS	Low score	**Ref**		**Ref**		**Ref**	
	Intermediate score	1.354 (1.272, 1.441)	1.87E-21	1.256 (1.179, 1.337)	1.72E-12	1.223 (1.139, 1.314)	3.26E-08
	High score	2.031 (1.803, 2.287)	1.39E-31	1.684 (1.492, 1.901)	3.07E-17	1.702 (1.487, 1.949)	1.27E-14
PSS_{CV} (linear)		1.176 (1.155, 1.197)	1.78E-72	1.144 (1.124, 1.165)	4.45E-49	1.138 (1.115, 1.162)	1.14E-35
PSS_{CV}	Low score	**Ref**		**Ref**		**Ref**	
	Intermediate score	1.258 (1.167, 1.357)	2.53E-09	1.195 (1.107, 1.29)	4.56E-06	1.17 (1.073, 1.275)	3.45E-04
	High score	1.817 (1.68, 1.965)	2.15E-50	1.643 (1.517, 1.779)	2.15E-34	1.606 (1.468, 1.757)	5.59E-25
**Seropositive RA**							
PSS (linear)		1.165 (1.088, 1.247)	1.23E-05	1.123 (1.047, 1.204)	1.17E-03	1.095 (1.011, 1.187)	0.027
PSS	Low score	**Ref**		**Ref**		**Ref**	
	Intermediate score	1.193 (1.034, 1.378)	0.016	1.138 (0.984, 1.317)	0.082	1.072 (0.909, 1.265)	0.408
	High score	1.773 (1.333, 2.358)	8.30E-05	1.564 (1.168, 2.093)	0.003	1.412 (1.006, 1.982)	0.046
PSS_{CV} (linear)		1.133 (1.086, 1.181)	6.01E-09	1.112 (1.065, 1.161)	1.15E-06	1.094 (1.042, 1.149)	3.18E-04
PSS_{CV}	Low score	**Ref**		**Ref**		**Ref**	
	Intermediate score	1.172 (0.985, 1.394)	0.074	1.141 (0.957, 1.36)	0.141	1.182 (0.969, 1.443)	0.099
	High score	1.616 (1.347, 1.939)	2.44E-07	1.51 (1.254, 1.818)	1.33E-05	1.425 (1.15, 1.765)	1.20E-03
