# Supplementary material for: Poor sleep and high rheumatoid arthritis risk: Evidence from large UK Biobank cohort
Source: PLoS One. 2025 Apr 23;20(4):e0318728. doi: 10.1371/journal.pone.0318728 (PMC12017501; doi:10.1371/journal.pone.0318728)
Supplement: S5 Table — Note: Model 1: Associations were adjusted for genotype batch, assessment centre, sex, TDI, age, kinship; Model 2: Associations were adjusted for genotype batch, assessment centre, sex, TDI, age, kinship, BMI, smoking status, alcohol; Model 3: Associations were adjusted for genotype batch, assessment centre, sex, TDI, age, kinship, BMI, smoking status, alcohol, diet, PA, DBP, SBP, HDL cholesterol, LDL direct, Cholesterol, Triglycerides, Cancer. Abbreviations: CI, confidence interval; HR, hazard ratio; TDI, Townsend deprivation index; BMI, body mass index; PA, physical activity; DBP, diastolic blood pressure; SBP, systolic blood pressure; HDL, high-density lipoprotein; LDL, low-density lipoprotein; PSS, poor sleep score. (PDF) [file pone.0318728.s010.pdf]

Poor sleep score	Model 1		Model 2		Model 3	
	**HR (95%CI)**	***P***	**HR (95%CI)**	***P***	**HR (95%CI)**	***P***
**RA**						
0	**Ref**		**Ref**		**Ref**	
1	1.102 (0.957, 1.267)	0.176	1.082 (0.939, 1.246)	0.276	1.116 (0.95, 1.31)	0.183
2	1.379 (1.204, 1.58)	3.65E-06	1.289 (1.123, 1.479)	2.96E-04	1.297 (1.109, 1.517)	1.15E-03
3	1.647 (1.431, 1.896)	3.67E-12	1.446 (1.253, 1.667)	4.20E-07	1.432 (1.216, 1.685)	1.61E-05
4~5	2.2 (1.862, 2.601)	2.29E-20	1.803 (1.521, 2.137)	1.06E-11	1.87 (1.543, 2.267)	1.74E-10
**Seropositive RA**						
0	**Ref**		**Ref**		**Ref**	
1	1.159 (0.84, 1.598)	0.369	1.12 (0.812, 1.546)	0.490	1.278 (0.874, 1.868)	0.206
2	1.282 (0.936, 1.755)	0.122	1.209 (0.882, 1.657)	0.237	1.279 (0.88, 1.859)	0.198
3	1.487 (1.072, 2.063)	0.017	1.338 (0.963, 1.861)	0.083	1.402 (0.949, 2.072)	0.090
4~5	2.04 (1.378, 3.02)	3.71E-04	1.755 (1.179, 2.613)	0.006	1.784 (1.115, 2.853)	0.016
