# Supplementary material for: Poor sleep and high rheumatoid arthritis risk: Evidence from large UK Biobank cohort
Source: PLoS One. 2025 Apr 23;20(4):e0318728. doi: 10.1371/journal.pone.0318728 (PMC12017501; doi:10.1371/journal.pone.0318728)
Supplement: S6 Table — Note: Associations were adjusted for age, sex, TDI, genotyping chip (UKB vs BiLEVE), and top 10 genetic PCs. Abbreviations: CI, confidence interval; HR, hazard ratio; GRS, genetic risk score; TDI, Townsend deprivation index; PC, principal components. (PDF) [file pone.0318728.s011.pdf]

	Cut-offs	HR (95% CI)	*P*
Training set	$<5\times10^{-4}$	1.122 (1.066-1.181)	9.96×10^{-6}
	$<5\times10^{-5}$	1.195 (1.112-1.285)	1.22×10^{-6}
	$<5\times10^{-6}$	1.270 (1.171-1.379)	1.03×10^{-8}
	**$<5\times10^{-7}$**	**1.281 (1.175-1.397)**	**2.22×10^{-8}**
	$<5\times10^{-8}$	1.437 (1.149-1.797)	1.47×10^{-3}
Test set	$<5\times10^{-4}$	1.145 (1.115-1.175)	1.06×10^{-24}
	$<5\times10^{-5}$	1.249 (1.205-1.295)	1.88×10^{-33}
	$<5\times10^{-6}$	1.315 (1.262-1.371)	1.58×10^{-38}
	**$<5\times10^{-7}$**	**1.330 (1.273-1.389)**	**2.69×10^{-37}**
	$<5\times10^{-8}$	1.295 (1.154-1.453)	1.05×10^{-5}
