# Supplementary material for: Poor sleep and high rheumatoid arthritis risk: Evidence from large UK Biobank cohort
Source: PLoS One. 2025 Apr 23;20(4):e0318728. doi: 10.1371/journal.pone.0318728 (PMC12017501; doi:10.1371/journal.pone.0318728)
Supplement: S7 Table — Note: Associations were adjusted for age, sex, TDI, genotyping batch, and top 10 genetic PCs. Abbreviations: CI, confidence interval; HR, hazard ratio; PC, principal components; GRS, genetic risk score. (PDF) [file pone.0318728.s012.pdf]

Sleep traits		GRS	All		Male		Female	
			HR (95%CI)	P	HR (95%CI)	P	HR (95%CI)	P
Sleeplessness	Never/rarely	Low GRS	**Ref**		**Ref**		**Ref**	
	Sometimes		1.166 (0.861, 1.579)	0.320	0.784 (0.513, 1.199)	0.262	1.884 (1.156, 3.073)	0.011
	Usually		2.017 (1.493, 2.724)	4.81E-06	1.259 (0.812, 1.953)	0.303	3.305 (2.037, 5.362)	1.29E-06
	Never/rarely	Intermediate GRS	1.512 (1.125, 2.034)	0.006	1.076 (0.735, 1.574)	0.707	2.405 (1.469, 3.936)	4.80E-04
	Sometimes		1.830 (1.393, 2.404)	1.43E-05	1.488 (1.052, 2.105)	0.025	2.681 (1.686, 4.264)	3.07E-05
	Usually		2.648 (2.010, 3.487)	4.30E-12	1.976 (1.379, 2.830)	2.04E-04	4.054 (2.548, 6.451)	3.49E-09
	Never/rarely	High GRS	2.019 (1.476, 2.763)	1.11E-05	1.495 (0.997, 2.240)	0.052	3.095 (1.849, 5.180)	1.71E-05
	Sometimes		2.650 (2.005, 3.501)	7.38E-12	1.926 (1.339, 2.769)	4.08E-04	4.123 (2.581, 6.585)	3.07E-09
	Usually		3.652 (2.752, 4.846)	2.94E-19	2.649 (1.813, 3.873)	4.87E-07	5.655 (3.532, 9.054)	5.40E-13
Sleep duration	7~8h	Low GRS	**Ref**		**Ref**		**Ref**	
	7h-		1.605 (1.281, 2.012)	3.95E-05	1.531 (1.040, 2.253)	0.031	1.660 (1.257, 2.193)	3.55E-04
	8h+		1.732 (1.251, 2.398)	9.28E-04	1.235 (0.671, 2.272)	0.498	2.022 (1.375, 2.974)	3.46E-04
	7~8h	Intermediate GRS	1.542 (1.320, 1.801)	4.75E-08	1.552 (1.199, 2.010)	8.55E-04	1.535 (1.263, 1.865)	1.59E-05
	7h-		2.032 (1.702, 2.426)	4.49E-15	2.118 (1.576, 2.845)	6.35E-07	2.015 (1.614, 2.515)	5.95E-10
	8h+		2.253 (1.794, 2.829)	2.71E-12	2.050 (1.391, 3.019)	2.82E-04	2.352 (1.775, 3.117)	2.67E-09
	7~8h	High GRS	2.109 (1.789, 2.485)	5.56E-19	2.158 (1.645, 2.832)	2.79E-08	2.078 (1.690, 2.554)	3.83E-12
	7h-		3.158 (2.616, 3.811)	4.26E-33	2.86 (2.066, 3.959)	2.40E-10	3.347 (2.656, 4.217)	1.24E-24
	8h+		2.888 (2.218, 3.761)	3.36E-15	2.246 (1.396, 3.613)	8.52E-04	3.237 (2.355, 4.450)	4.66E-13
Snoring	No	Low GRS	**Ref**		**Ref**		**Ref**	
	Yes		1.239 (0.997, 1.539)	0.053	1.237 (0.862, 1.775)	0.249	1.280 (0.970, 1.689)	0.081
	No	Intermediate GRS	1.499 (1.280, 1.754)	4.66E-07	1.710 (1.272, 2.297)	3.75E-04	1.418 (1.178, 1.708)	2.31E-04
	Yes		1.644 (1.384, 1.951)	1.39E-08	1.765 (1.308, 2.382)	2.01E-04	1.592 (1.285, 1.973)	2.11E-05
	No	High GRS	2.161 (1.832, 2.549)	5.99E-20	2.295 (1.680, 3.134)	1.78E-07	2.107 (1.734, 2.561)	6.35E-14
	Yes		2.306 (1.918, 2.773)	6.07E-19	2.422 (1.766, 3.320)	3.94E-08	2.281 (1.807, 2.878)	3.72E-12
Morning/evening	Definitely morning	Low GRS	**Ref**		**Ref**		**Ref**	
	Morning more		1.081 (0.823, 1.420)	0.576	0.924 (0.581, 1.471)	0.740	1.171 (0.836, 1.641)	0.359
	Evening more		1.013 (0.754, 1.360)	0.933	0.912 (0.554, 1.501)	0.717	1.070 (0.743, 1.543)	0.715
	Definitely evening		1.110 (0.729, 1.689)	0.628	0.807 (0.372, 1.752)	0.588	1.296 (0.785, 2.141)	0.311
	Definitely morning	Intermediate GRS	1.524 (1.198, 1.939)	6.04E-04	1.516 (1.016, 2.262)	0.042	1.530 (1.132, 2.069)	0.006
	Morning more		1.377 (1.087, 1.743)	0.008	1.214 (0.816, 1.805)	0.338	1.468 (1.094, 1.971)	0.010

	Evening more		1.724 (1.360, 2.186)	6.85E-06	1.733 (1.171, 2.566)	0.006	1.718 (1.275, 2.315)	3.77E-04
	Definitely evening		1.574 (1.166, 2.125)	0.003	1.502 (0.912, 2.473)	0.110	1.620 (1.112, 2.359)	0.012
	Definitely morning	High GRS	2.045 (1.583, 2.642)	4.43E-08	1.840 (1.195, 2.834)	0.006	2.162 (1.573, 2.974)	2.08E-06
	Morning more		2.051 (1.606, 2.619)	8.56E-09	1.916 (1.274, 2.882)	0.002	2.117 (1.560, 2.874)	1.50E-06
	Evening more		2.268 (1.764, 2.916)	1.67E-10	1.811 (1.179, 2.783)	0.007	2.543 (1.864, 3.470)	3.91E-09
	Definitely evening		2.410 (1.741, 3.336)	1.15E-07	2.173 (1.252, 3.770)	0.006	2.564 (1.713, 3.837)	4.69E-06
Daytime dozing	Never/rarely	Low GRS	**Ref**		**Ref**		**Ref**	
	Sometimes		1.265 (1.000, 1.601)	0.050	1.128 (0.752, 1.693)	0.561	1.351 (1.012, 1.803)	0.041
	Usually		1.526 (0.891, 2.613)	0.124	1.909 (0.883, 4.125)	0.100	1.266 (0.595, 2.695)	0.541
	Never/rarely	Intermediate GRS	1.496 (1.301, 1.721)	1.71E-08	1.603 (1.260, 2.040)	1.24E-04	1.444 (1.216, 1.715)	2.86E-05
	Sometimes		1.617 (1.356, 1.928)	8.74E-08	1.627 (1.213, 2.181)	0.001	1.613 (1.294, 2.011)	2.16E-05
	Usually		2.236 (1.608, 3.109)	1.73E-06	2.145 (1.257, 3.660)	0.005	2.303 (1.514, 3.504)	9.72E-05
	Never/rarely	High GRS	1.986 (1.711, 2.305)	1.77E-19	2.036 (1.575, 2.632)	5.85E-08	1.959 (1.631, 2.352)	5.75E-13
	Sometimes		2.639 (2.192, 3.176)	1.16E-24	2.333 (1.695, 3.211)	2.00E-07	2.834 (2.257, 3.559)	3.21E-19
	Usually		3.805 (2.672, 5.416)	1.22E-13	4.063 (2.347, 7.032)	5.49E-07	3.620 (2.278, 5.752)	5.23E-08
