# Supplementary material for: Poor sleep and high rheumatoid arthritis risk: Evidence from large UK Biobank cohort
Source: PLoS One. 2025 Apr 23;20(4):e0318728. doi: 10.1371/journal.pone.0318728 (PMC12017501; doi:10.1371/journal.pone.0318728)
Supplement: S8 Table — Note: # represents the utilization of linear regression due to the continuous Abbreviations: CI, confidence interval; PSS, poor sleep score; CV, cross-validation. (PDF) [file pone.0318728.s013.pdf]

Sleep phenotypes	Estimate (95%CI)	***P***
Getting up	1.293 (1.156, 1.446)	6.82E-6
chronotype	1.072 (0.962, 1.193)	0.207
Nap during day	1.088 (0.967, 1.223)	0.161
Insomnia	1.027 (0.920, 1.148)	0.633
Daytime dozing	1.083 (0.945, 1.242)	0.250
Snoring	1.015 (0.895, 1.151)	0.819
Sleep duration[#]	0.021 (-0.046, 0.087)	0.542
PSS[#]	0.191 (0.095, 0.288)	1.00E-4
PSS (three categories)	1.199 (1.073, 1.338)	1.28E-3
PSS_{CV}[#]	0.289 (0.143, 0.434)	1.05E-4
PSS_{CV} (three categories)	1.170 (1.048, 1.306)	0.005
